# Primary aneurysmal bone cyst of patella

**DOI:** 10.4103/0019-5413.50859

**Published:** 2009

**Authors:** N Somasekhar Reddy, Venkata Reddy Sathi

**Affiliations:** Department of Orthopaedics Apollo Hospitals, Hyderabad, India

**Keywords:** Aneurysmal bone cyst, patella, curettage, bone grafting

## Abstract

Of all the aneurysmal bone cysts (ABC) occurring in the body, less than 1% are seen in the patella. We report here, a 27-year-old woman with Stage III ABC of patella. Curettage, chemical and thermal cautery of the bed followed by autogenous bone grafting of the defect was done. At two-year follow up, there was a suspicion of lucency in the middle of the patella. However a repeat curettage revealed only fibrous tissue. Now at four years of follow up, the bone graft remained well incorporated. Patient has mild anterior knee pain on stair climbing but regained normal knee function.

## INTRODUCTION

Jaffe and Lichtenstein^1^ described aneurysmal bone cyst as a peculiar blood-containing cyst of large size in 1942. The lesions were expansile and showed evidence of erosion of the surrounding bone and encroachment of the surrounding tissues. Upon surgical exposure of the lesions, a thin, bony wall that contained bloody fluid was found.^1^

The aneurysmal bone cysts (ABC) are generally considered rare, accounting for only 1%–6% of all primary bony tumors.^2^ Less than 1% of all cases of ABCs occur in the patella. ABC most often affects individuals during their second decade of life and may occur in any bone in the body. Although benign, the ABC can be locally aggressive and can cause extensive weakening of the bony structure and impinge on the surrounding tissues. The true etiology and pathophysiology remain a mystery, but the mainstay of treatment has been intralesional curettage. Recurrence is not uncommon. Other surgical options include en bloc resection or wide excision, selective arterial embolization and curettage with locally applied adjuvants such as liquid nitrogen or phenol.

## CASE REPORT

In June 2003, a 27-year-old woman with no history of trauma presented with complaints of intermittent pain and swelling in the anterior aspect of the left knee for 9 months, with increased intensity of pain for 3 months. A physical examination revealed a tender, soft, cystic swelling in the anterior aspect of the knee over the patella, with full range of movements.

**Figure 1:**
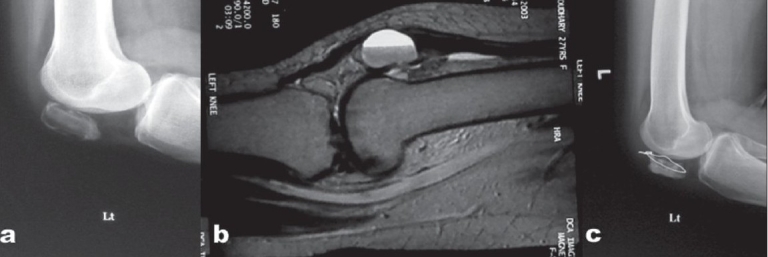
X-ray of left knee (lateral view) showing stage 3 ABC in left patella (a) T_2_ weighted saggital MRI of left knee showing fluid level in the cystic patella (b) Two-year follow-up X-ray showing well formed patella but with central lucency (c)

Lateral radiograph [Figure 1a] showed an osteolytic lesion occupying almost the entire patella with cortical thinning. Magnetic resonance images showed a large cyst with fluid level in the patella [Figure 1b]. It also confirmed loss of cortical bone over the anterior patellar surface. Patient was advised excision of the remaining shell of the patella. But patient was against patellectomy and preferred a reconstructive procedure accepting the risk of recurrence. At surgery, through mid-line approach, quadriceps tendon was incised vertically directly into the cyst which had no anterior cortical wall. Only a rim of cortical bone was found at the medial and lateral borders of the patella with some bony islands at the attachments of quadriceps and patellar tendons. The subchondral bone was also found to be very thin after curettage. Thermal and chemical cauterization with phenol was also performed. Autogenous bone graft was used to fill the defect. Circlage wiring was done to protect the remaining bony shell [Figure 1c]. Histopathological examination confirmed the diagnosis. Postoperatively the knee was immobilized in a cylindrical cast with knee in 15° of flexion for 4 weeks followed by full weight bearing and joint mobilization exercises.

At two-year follow-up, there was a suspicion of lucency in the middle of the patella. Fearing a recurrence, patellectomy was advised again. However, as the patient was reluctant, a repeat curettage was done along with removal of the circlage wire. Biopsy of the material revealed only fibrous tissue. Now at four years of follow-up, the bone graft remained well incorporated with no signs of recurrence.

## DISCUSSION

Aneurysmal bone cysts like other benign lesions were staged into three groups by Enneking. Stage 1 lesions have a well defined cortex. Stage 2 lesions have a thinned cortex, which may be partly broken but limited to the periosteum. Stage 3 lesions penetrate the cortex with small breaches around the perimeter. Our patient had a stage 3 lesion. Patellectomy is indicated in stage 3 lesions.^3^ However, at the insistence of our patient, we attempted a reconstructive procedure to salvage the patella. So we believe that in some cases with stage 3 disease, a salvage can be attempted if the patient accepts the risk of recurrence needing subsequent patellectomy.
